# Gallbladder protrusion through the groin region—a very unusual femoral hernia

**DOI:** 10.1259/bjrcr.20180035

**Published:** 2018-08-11

**Authors:** Lucas Kreutz Rodrigues, Rebeca Bosse De Jesus, Giovanni Brondani Torri, Marcos Momolli, Leonardo Kreutz Rodrigues, Caroline Lorenzoni Almeida Ghezzi, Leandro Totti Cavazzola

**Affiliations:** 1 Department of General Surgery, Hospital de Clínicas de Porto Alegre, Porto Alegre, RS, Brazil; 2 Department of Vascular Surgery, Hospital de Clínicas de Porto Alegre, Porto Alegre, RS, Brazil; 3 Department of Radiology and Imaging, Hospital de Clínicas de Porto Alegre, Porto Alegre, RS, Brazil; 4 Medical School of Medicine, Universidade Federal de Santa Maria, Santa Maria, RS, Brazil

## Abstract

Groin hernias are among the oldest recorded afflictions of mankind. Most of them protrude through the inguinal canal, and only a few through the femoral canal. Usually, they are present as a painful lump in the groin region, and their complications arise if they become incarcerated or strangulated. Incarcerated hernias may contain a variety of contents, such as the omentum, small bowel, colon, bladder, appendix, stomach, or ovary as previously described. Usually, the history and a physical examination are sufficient to make the diagnosis. However, the wide use of CT has become an effective instrument to identify the contents of hernias and has helped surgeons program the best management. This article reports, for the first time, the case of an 81-year-old female with an incarcerated femoral hernia that contains the gallbladder.

## Case report

**Figure 1.**
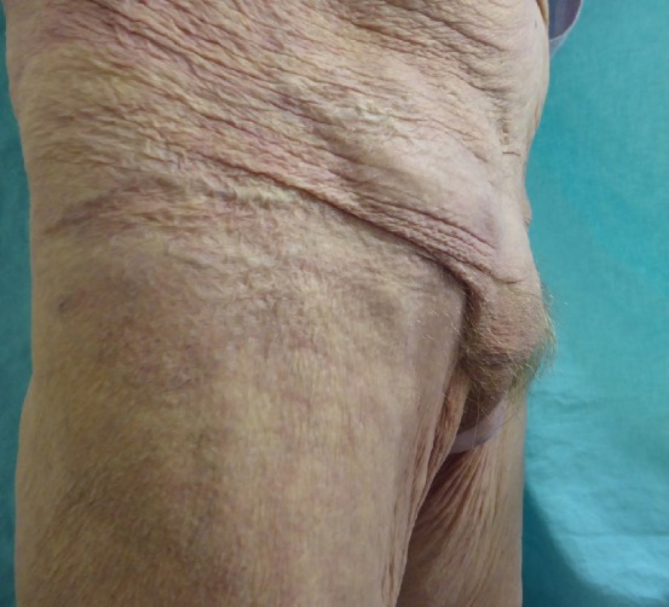
Right groin bulge and cystocele seen in patient standing.

An 81-year-old Caucasian female, healthy weight (44 kg, or 97 pounds), without systemic diseases, presented herself in a tertiary hospital ambulatory with a complaint of bulge and pain in the right groin for 10months. The pain was mild and usually appeared when she performed physical effort. No further symptoms were recorded. The patient had no episode of acute cholecystitis previously. She had urinary incontinence surgery 12 years ago and a Lichtenstein hernioplasty on the left side 10 years ago with no signs of recurrence. She had a descending thoracic aortic aneurysm measuring 7.1×6.3 cm on its major axial diameter and an infrarenal abdominal aortic aneurysm measuring 6.4×6.1 cm on its axial diameter and was planning to undergo an endovascular repair in two steps. She also had a cystocele. The physical exam showed a bulge on the right inguinal region with no expansion on coughing. The palpation showed a hard bulge and the hernia was not reducible with pain on manipulation (Figure 1).

## Investigators

**Figure 2.**
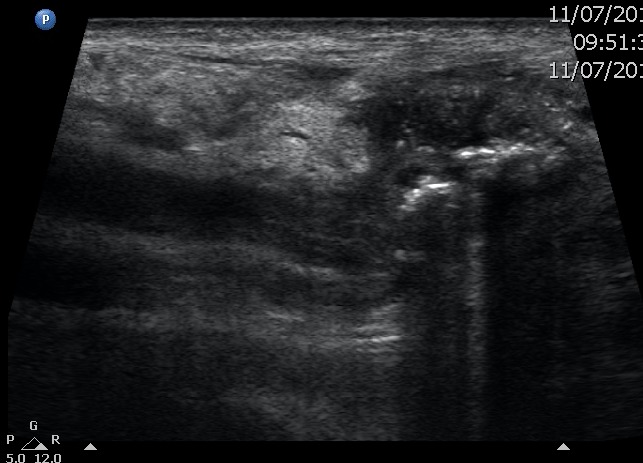
Numerous small calculi with posterior acoustic shadow.

**Figure 3.**
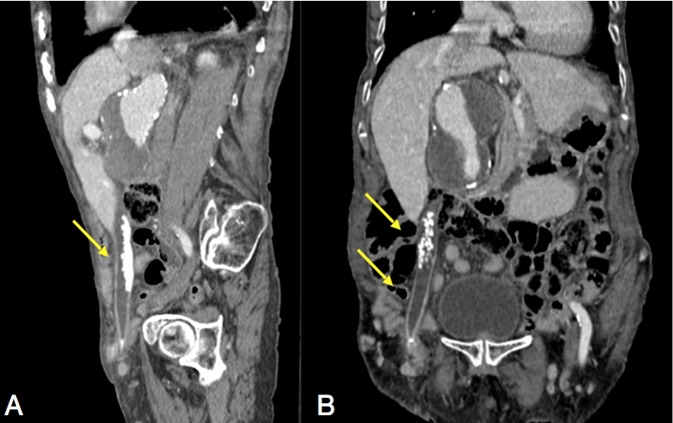
Abdominal CT in the sagittal (A) and coronal (B) axis showing the gallbladder containing various calculi and extending through the femoral canal (arrows).

**Figure 4.**
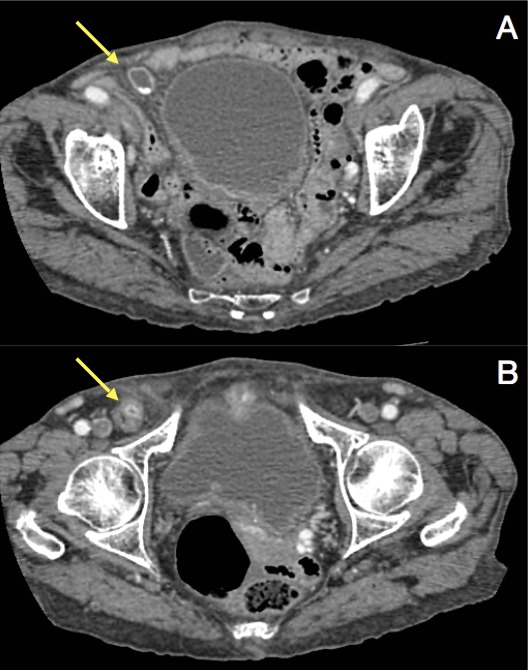
CT of the pelvis in the axial plane shows (A) the gallbladder body medially to the right external iliac vessels (arrow) and (B) the gallbladder fundus in the femoral region (arrow).

An ultrasound was performed and found an elongated tubular structure with numerous calculi coming from the right hypochondrium and projecting into the femoral canal (Figure 2). There was no sign of parietal thickening (gallbladder wall: 0.3 cm) although a thin laminar liquid collection was observed around the gallbladder fundus. A CT was ordered and confirmed the presence of a tubular-shaped gallbladder, with a length of 23.2 cm and volume of 33.8 cm³ coursing inferiorly to the inguinal region, medially to the inferior epigastric vessels and extending 3.5cm into the femoral canal, medially to the right common femoral vein (Figures 3 and 4). The patient had no signs of other hepatobiliary abnormalities. The liver did not present any abnormality, with a volume estimated at 1041 cm^3^, with no sign of biliary dilation. The common hepatic and common bile duct had a length of 6.4 cm and caliber of 0.5 cm.

## Differential diagnosis

History and physical examination are the main means of diagnosing groin hernias. Some differential diagnoses include: groin abscess, hematoma, lipoma, lymphadenitis, pseudoaneurysm, tumor and in males, retracted testes, testicular torsion or acute epididymitis. After an ultrasound or CT, the diagnosis of hernia becomes evident.

## Treatment

After endovascular elective surgery, an elective laparoscopic cholecystectomy followed by a transabdominal preperitoneal patch plasty of the femoral hernia repair were planned.

## Literature review

### Search strategy

An extensive PUBMED, MEDLINE and Scielo literature search was performed using the MeSH terms “inguinal hernia”, “femoral hernia” and “gallbladder”, on January 17, and 47 articles were found; 2 of them were case reports of inguinal hernia with gallstones. Following searches in PUBMED and web searches on Google with the MeSH terms “Hernia” and “Gallbladder”, 363 results were found. Four of them described incisional hernia which contains the gallbladder. Four paraostomal gallbladder hernia articles were also found, while the description of ventral herniation of gallbladder was less frequent. The most infrequent was the Spigelian hernia, in which the gallbladder bulges out through the semilunar line. In the research, no previous reports of gallbladder herniation through the femoral canal were found.

## Discussion

Groin hernia is a very frequent cause of outpatient visits in surgical units. The prevalence of groin hernia in the USA is estimated to be about 5–10%.^1^ However, of all groin hernias, 96% are inguinal and 4% are femoral.^2^ These hernias are far more frequent in females, unlike inguinal hernia, particularly older females. The main reason is the wider shape of the female pelvis.^3^ The femoral hernia protrudes through the femoral ring and reaches the femoral canal, which is bordered anterosuperiorly by the inguinal ligament, posteriorly by the pectineal ligament, medially by the lacunar ligament and laterally by the femoral vein. It is present as a painful lump in the inner upper part of the thigh or groin that can frequently be pushed back in or bulge depending on intra-abdominal pressure.^4^ It originates through a weak spot in the surrounding muscle wall in the femoral canal. The history and physical examination are usually sufficient to make the diagnosis.^5^ There is a need to be aware of the potential contents of hernia sacs, which has implications for definitive operative management.^6^ Gallbladder herniation is rare and may occur internally through the foramen of Winslow^7^ or in the subcutaneous tissue through a defect on the abdominal wall.^8, 9^ This case report describes the case of an 81-year-old female with an inguinal hernia whose symptoms started 1 year ago. Females tend to develop this type of condition older than males. In one review, the median age for groin hernia presentation was from 60to79years old for women.^10^ The treatment of femoral hernia is surgical, independent of which structure protrudes through it, in order to relieve symptoms and avoid complications such as obstruction or strangulation. The patient was very symptomatic from her right groin. However, the thoracic and the abdominal aorta aneurysms were the priorities and planned to be treated earlier by endovascular approach. A laparoscopic cholecystectomy and inguinal repair was planned to be performed subsequently.

**Figure 5.**
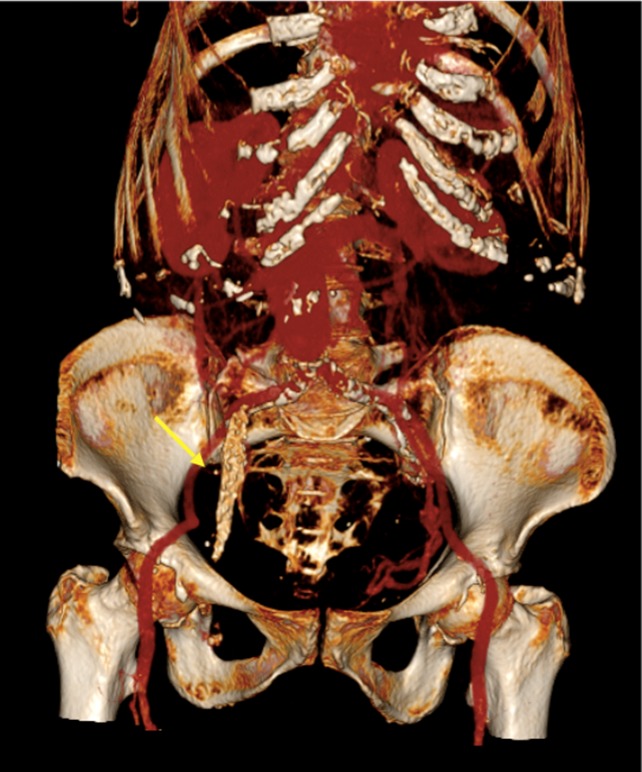
Three-dimensional reconstruction of CT of the abdomen showing a gallbladder full of calculi extending to the femoral region (arrow).

Our main intention with this case report was to describe, for the first time, a femoral hernia where its content is the gallbladder (Figure 5). Hernias are among the oldest recorded afflictions of mankind.^11^ Surgeons have been exposed to these conditions for a very long time and it has become a tradition, mainly with inguinal hernias, whenever they identify a new structure in the hernia sac, to describe them with their name.^12^ It has been done by several surgeons; Alexander Littré, in 1700, named an inguinal hernia which had a meckel diverticulum inside it, Rene Jacques Garengeot identified, in 1721, a cecal appendix in a femoral hernia, Claudius Amyand, in 1735, faced an acute appendicitis inside the inguinal canal and August Richter, in 1778, described a hernia in which the antimesenteric wall of the intestine protruded through a defect in the abdominal wall. At the time, abnormal findings inside hernia sacs were discovered during the intraoperative process, and this is the reason why rare hernias were described by surgeons. However with the use of imaging exams, it is possible to make an early diagnosis and be better prepared for the procedure. Almost 300years after these records, this case report identified a femoral hernia in which the gallbladder protrudes through the inguinal canal, which had never been described before. It is important to believe that despite all the improvements and new findings in Medicine in recent years, this science still has anatomical and surgical discoveries to be done.

## Learning points

- The case presented illustrates a different type of femoral hernia in which its content is the gallbladder.
- Whenever there are new or abnormal findings on physical examination, imaging exams (Ultrasound or CT) should be requested. The patient had a hard bulge in the groin region due to gallstones.
- Although it is unusual, Medicine still has anatomical findings to be done.
